# Cell functional enviromics: Unravelling the function of environmental factors

**DOI:** 10.1186/1752-0509-5-92

**Published:** 2011-06-06

**Authors:** Ana P Teixeira, João ML Dias, Nuno Carinhas, Marcos Sousa, João J Clemente, António E Cunha, Moritz von Stosch, Paula M Alves, Manuel JT Carrondo, Rui Oliveira

**Affiliations:** 1 Instituto de Tecnologia Química e Biológica - Universidade Nova de Lisboa (ITQB-UNL), Av. República, Quinta do Marquês, 2781-901 Oeiras, Portugal; 2 Instituto de Biologia Experimental e Tecnológica (IBET), Av. República, Quinta do Marquês, 2781-901 Oeiras, Portugal; 3 REQUIMTE, Systems Biology & Engineering Group, Departamento de Química, Faculdade de Ciências e Tecnologia, Universidade Nova de Lisboa (FCT-UNL), 2829-516 Caparica, Portugal

## Abstract

**Background:**

While functional genomics, focused on gene functions and gene-gene interactions, has become a very active field of research in molecular biology, equivalent methodologies embracing the environment and gene-environment interactions are relatively less developed. Understanding the function of environmental factors is, however, of paramount importance given the complex, interactive nature of environmental and genetic factors across multiple time scales.

**Results:**

Here, we propose a systems biology framework, where the function of environmental factors is set at its core. We set forth a "reverse" functional analysis approach, whereby cellular functions are reconstructed from the analysis of dynamic envirome data. Our results show these data sets can be mapped to less than 20 core cellular functions in a typical mammalian cell culture, while explaining over 90% of flux data variance. A functional enviromics map can be created, which provides a template for manipulating the environmental factors to induce a desired phenotypic trait.

**Conclusion:**

Our results support the feasibility of cellular function reconstruction guided by the analysis and manipulation of dynamic envirome data.

## Background

The phenotype of a cell results from the combined effect of environmental and genetic factors [1,2]. While the investigation of the relationship between genetic and environmental factors at the molecular level has proved difficult [3], a number of recent systems biology studies have produced important insights about the nature of this complex relationship.

The relationship between genes and environment can be analysed by computational methods founded on the principle that the biochemical habitat of a cell is primarily "engraved" in its DNA sequence. Genes may be associated with metabolic enzymes, membrane transporters, signal transduction or regulatory control. Combined with basic biochemical information currently available in several databases (e.g. KEGG [4] and BioCyc databases [5]), it is possible to reconstruct the majority of the metabolic reaction network and also the associated exo-metabolome [6]. This is the currently accepted top-down, gene-to-function molecular biology paradigm.

Following these principles, Borenstein et al., [7] applied a graph-theoretical algorithm to determine these exogenously acquired compounds - they called it the seed set of an organism - for each of the 478 prokaryotic species with available metabolic networks in the KEGG database [4]. They found that about 8-11% of the compounds in the metabolic network of an organism correspond to the seed set and that each organism possesses a characteristic seed set. Moreover, comparing the seed set of the different organisms enabled them to trace the evolutionary history of both metabolic networks and growth environments across the tree of life, supporting the "reverse" ecology principle.

Using comparative genomics and flux balance analysis, Pal *et al*. [8] concluded that the adaptive evolution of bacterial metabolic networks in response to changing environments proceeds essentially by horizontal gene transfer (i.e. genes acquired from other species) of genes involved primarily in the transport and catalysis of external nutrients. With a similar approach, Kreimer *et al*. [9] studied the modularity of metabolic networks among 325 prokaryotic species with sequenced genomes. They observed that several environmental factors contributed to the variation in metabolic-network modularity across species. Their observations corroborated the findings of Pal *et al*. [8] that the variability in the natural habitat of an organism promotes modularity in its metabolic network.

Allen *et al*. [10] and Kell *et al*. [11] analysed the complete set of metabolites excreted or secreted by yeast cultures using high-throughput direct injection mass spectrometry. They called the technique "metabolic footprinting". They observed that a high number of metabolites, typically between 50-150 metabolites, are either excreted or secreted to the medium. Using statistical methods they showed that the information contained in the environment is sufficient to distinguish between different physiological states of wild type yeast strains and single-gene deletion mutants and concluded that the technique has high potential for functional analysis and metabolic engineering.

All the above studies concur with a profound, bi-directional link between environment and genes, with routes on the evolutionary principles of life. When exposed to a particular environment, a given genotype will respond and change its environment in a unique way, which can be distinguished even between single-gene deletion mutants [10]. Supported by these observations, we explored the feasibility of a bottom-up, envirome to cellular function reconstruction approach. Instead of using transcriptome data as in functional genomics, we set out to determine if it is possible to reconstruct cellular function from the analysis of envirome data sets alone. We term this reverse functional analysis approach "cell functional enviromics". In what follows, we describe the principles of the employed methodology and show how this approach can be applied to reconstruct Baby Hamster Kidney (BHK) metabolism.

## Results and Discussion

### A systems biology approach to cell functional enviromics

In functional genomics, the goal is genome-wide cellular function reconstruction through the collection and analysis of transcriptome or proteome data over time. In functional enviromics, we attempt to bridge environmental factors and function through the collection and analysis of dynamic envirome data. The necessary elements to such an analysis are: **i) **setting the universe of cellular functions and envirome components, **ii) **collecting informative envirome data over time, and **iii) **systems level analysis of dynamic envirome data to find relationships between environmental variables and cellular functions.

To support such a framework, we have developed a computational algorithm called "envirome-guided projection to latent pathways (PLP)". "Cellular function" has been defined in different ways, here we adopted elementary flux modes (EM) as function descriptors [12-18] because they enable systematic, large-scale computational analysis of biochemical networks from a functional viewpoint. Mathematically, EMs form the convex basis of the null space solution of a metabolic network stoichiometric matrix. Biologically, they represent elementary network topologies defining all possible independent operational modes of a cell. EMs have been previously used to support computational functional genomics [19,20]. Here we apply the same rational to derive a functional enviromics algorithm.

A medium scale network has typically a very large number of EMs in the order of ~10^6 ^[21]. It is however unlikely that all of these cellular functions are active at the same time. In reality, despite the apparent complexity of cellular networks, only a very limited number of core network topologies are capable of robustly executing any particular biological function [22]. It is thus rational to assume that a given environmental stimulus activates only a moderate number of characteristic EMs.

**Figure 1:**
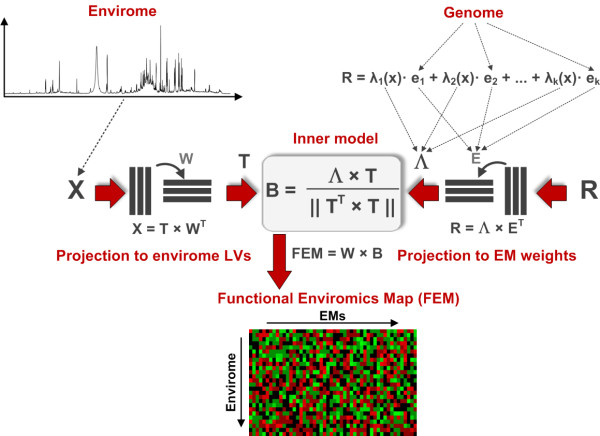
**Schematic representation of a cell functional enviromics algorithm**. The aim is to maximise the covariance between measured environmental factors (X) and rate of change of environmental factors (R) under the constraint of known genes translated into a plausible set of elementary flux modes (E). As in conventional partial least squares, the input matrix (X) is decomposed into a loadings matrix of latent variables (W) and a scores matrix (T). The response data (R), however, are decomposed into genome dependent factors (the structure of elementary flux modes, E) and envirome dependent factors (weights of elementary flux modes, Λ). Then, only the envirome dependent factors (Λ) are linearly regressed against envirome data (X). Finally, such regression coefficients are organized into a functional enviromics map (FEM) with columns representing EMs, rows environmental factors and each element representing the strength of up- or down-regulation of each core cellular function by each envirome factor. Mathematical details can be found as Methods.

In conformity with the above enumerated principles, PLP was designed to maximise the covariance between environmental state (envirome data sets) and observed phenotypic trait (rate of change of envirome variables) under the constraint of known genes translated into a plausible set of cellular functions (Figure 1). Simply put, PLP implements a discrimination algorithm to find a minimal set of EMs based on two criteria: **i) **variance of measured phenotypic trait explained by each EM and **ii) **degree of correlation of each EM with the environmental state. By maximising these two criteria, the algorithm delivers a ranking of EMs in increasing order of percentage of explained variance in the measured flux data, and a functional enviromics map (FEM) representing the strength of up- or down-regulation of EMs by environmental factors. Mathematical details of the method can be found in the Methods section. In what follows we illustrate the methodology with an application to a recombinant BHK cell line expressing the fusion glycoprotein IgG1-IL2 (see Methods for details).

### Envirome guided metabolic reconstruction of BHK cells

**Figure 2:**
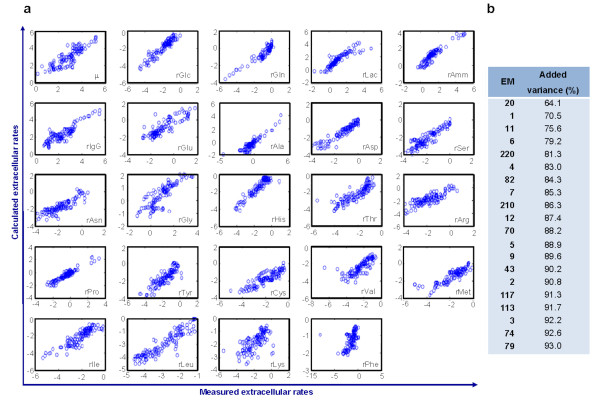
**Application of the PLP algorithm to the analysis of envirome data collected from six BHK cultures as described in Methods and Table 1**. Data from the growth stage of every experiment were pooled together. (**a**) Correlation between measured extracellular rates (R) and predicted rates obtained by reconstruction of fluxes of discriminated EMs. Units are in nmol.(10^6^cells.h)^-1^. (**b**) Ranking of first 20 EMs that correlate with the envirome, explaining 93% of the variance in the measured extracellular rates. These pathways correspond to typical routes known to be active in cultured mammalian cell lines. Refer to the text for a description.

The methodology was first applied to the analysis of a data set comprising measurements of 27 environmental factors collected from six independent, differently operated culture experiments as described in Methods and Table 1. The main objective was to identify the "active" set of EMs by iteratively projecting the metabolism into a minimal set of core cellular functions that correlate with the envirome. The predictor (input) matrix included an extensive list of environmental factors including: temperature, pH, osmolality and concentrations of 24 measured components (viable cells, glucose, lactate, ammonia, glycoprotein and 19 amino acids). The target (output) matrix consisted of the 24 exchange fluxes calculated from the profiling of extracellular concentrations. The results are shown in Figure 2. The explained flux variance was calculated according to Eq. (4). It represents the variance in the flux data that is explained by a model built on the selected EMs.

**Table 1:** Operating conditions of the fed-batch experiments

		Fed-batch 1	Fed-batch 2	Fed-batch 3	Fed-batch 4	Fed-batch 5
**Initial concentration (mM)**	**Glc**_**i**_	5.5	5.7	0.18	6.2	4.8
	**Gln**_**i**_	0.2	0.5	0.42	3.9	0.1
	**Glu**_**i**_	1.6	0.6	0.82	0.6	4.0
	**Ser**_**i**_	2.3	1.3	1.30	1.4	1.4
	**Asp**_**i**_	1.4	0.8	0.65	0.8	0.8

	**First feed** Started when Glc and/or Gln went below 0.15 mM	Glc = 183.0 Gln = 15.0 Glu = 20.0	Glc = 182.0 Gln = 5.2 Glu = 31.0	Glc = 150.0 Gln = 4.2 Glu = 29.2	Glc = 198.0	Glc = 183.0
	
**Composition of feeds (mM)**	**Second feed** Started when Ser became limiting (~75 h of operation)	-	Glc = 193.0 Gln = 4.5 Glu = 36.0 Ser = 33.0 Cys = 8.5	Glc = 160.0 Gln = 5.4 Glu = 25.6 Culture medium 5 times concentrated	Glc = 156.0 Gln = 42.8	-

**Flow rate**	Adjusted to keep glucose between 0.05-0.15 mM or/and glutamine between 0.05-0.25 mM					

A high degree of correlation was observed between measured envirome components and relative weight of discriminated EMs, ultimately reflected in high correlation between measured and predicted extracellular rates (Figure 2a). Within the universe of 251 possible EMs, 20 of them captured up to 93% of the measured rates variance (Figure 2b), even though the data were pooled together from several independent cultivation experiments, with inherently diverse environmental trajectories. The first selected EM is one of many for biomass synthesis (see Tables 2 and 3), describing more than 64% of total rate data variance. This concurs with the fact that most of the processed carbon is involved in biomass synthesis. The single EM for product formation (EM 1) is also selected, explaining part of the specific consumption rates of every amino acid. The EM corresponding to anaerobic conversion of glucose into lactate was selected in third place (EM 11). Given the low saturation constants of glucose transporters (Km = 1-2 mM) [23,24]), this pathway usually carries a high flux in mammalian cell cultures, leading to waste lactate formation. Serine transamination into glycine was the fourth selection (EM 6). Indeed, the rates of serine consumption and glycine production were quite high, mainly during the initial periods of culture. This pathway may represent a strategy for ammonia detoxification, the main toxic by-product in mammalian cell culture. The sixth position (EM 4) corresponds to the pathway of glutaminolysis, well known as a major carbon source for energy production in mammalian cells [25,26]. Complete oxidation of glucose in the TCA cycle (EM 43) is also selected, reflecting the metabolic shift induced during glucose fed-batch experiments.

**Table 2:** Ranking of statistically significant EMs for sequential metabolic phases of fed-batch 3

Phase I		Phase II		Phase III		Phase IV		Phase V	
**EM**	**Var (%)**	**EM**	**Var (%)**	**EM**	**Var (%)**	**EM**	**Var (%)**	**EM**	**Var (%)**

110	61.76	250	65.02	62	47.28	62	80.69	62	84.30
**6**	**69.92**	**1**	**89.68**	**1**	**90.61**	12	85.98	**6**	**89.43**
**11**	**75.82**	41	93.81	**11**	**92.80**	**1**	**89.37**	**1**	**94.44**
**-7**	**81.63**	28	96.57	**6**	**93.96**	86	91.08	49	97.04
2	87.34	**43**	**97.04**	80	95.61	**5**	**92.29**	201	97.53
**5**	**91.93**	**-4**	**97.40**	**5**	**96.28**	**43**	**93.11**	119	97.84
**4**	**94.05**	**5**	**97.63**	**43**	**96.86**	**7**	**93.48**	**-4**	**98.14**
101	95.86	47	97.84	**7**	**97.07**	**6**	**93.88**	**11**	**98.43**
**43**	**96.45**	**6**	**98.01**	201	97.14	69	94.00	**43**	**98.67**
**1**	**97.02**	**11**	**98.13**	**-4**	**97.22**	**11**	**94.12**	102	98.87
86	97.55	80	98.24	3	97.27	225	94.16	**5**	**98.95**
45	97.67	102	98.29	45	97.31	**-4**	**94.19**	**7**	**99.03**
33	97.77	15	98.31	102	97.34	102	94.27	87	99.11
24	97.83	**7**	**97.34**	86	97.35	79	94.30	15	99.15
41	97.89	87	97.35	81	97.36			37	99.23
74	97.91	10	97.36	88	97.37			14	99.27
3	97.92	9	97.37						

The minus sign indicates flux in the opposite direction for those EMs whose participating reactions are all reversible. EMs in bold were conserved between cultures and also selected from the full data set (see Figure 2b), showing the ability of the PLP algorithm to identify essential common features in metabolism.

**Table 3:** Stoichiometry of macroscopic reactions obtained from the elementary flux modes discriminated in Figures 2b and Tables 2, 5 and 5

**EM 1**	0.052 Gln + 0.048 Glu + 0.034 Ala + 0.033 Asp + 0.073 Ser + 0.035 Asn + 0.028 Gly + 0.026 His + 0.065 Thr + 0.049 Arg + 0.073 Pro + 0.069 Tyr + 0.016 Cys + 0.081 Val + 0.015 Met + 0.037 Ile + 0.109 Leu + 0.081 Lys + 0.065 Phe → IgG1-IL2
**EM 2**	His → Glu + Amm
**EM 3**	Phe → Tyr
**EM 4**	Gln → Glu + Amm
**EM 5**	Glu → Pro
**EM 6**	Ser + Amm → 2 Gly
**EM 7**	Asn → Asp + Amm
**EM 9**	Glu → Ala + 5 CO_2_
**EM 11**	Glc → 2 Lac
**EM 12**	Ser → Lac + Amm
**EM 19**	413 Glc + 311 Gln + 213 Ala + 215 Asp + 183 Ser + 114 Asn + 253 Gly + 56 His + 148 Thr + 153 Arg + 136 Pro + 67 Tyr + 72 Cys + 155 Val + 65 Met + 109 Ile + 209 Leu + 175 Lys + 82 Phe → Biomass + 23 Glu
**EM 20**	126 Glc + 311 Gln + 213 Ala + 215 Asp + 758 Ser + 114 Asn + 253 Gly + 56 His + 148 Thr + 153 Arg + 136 Pro + 67 Tyr + 72 Cys + 155 Val + 65 Met + 109 Ile + 209 Leu + 175 Lys + 82 Phe → Biomass + 23 Glu + 575 Amm
**EM 21**	126 Glc + 311 Gln + 213 Ala + 215 Asp + 183 Ser + 114 Asn + 253 Gly + 56 His + 148 Thr + 153 Arg + 136 Pro + 67 Tyr + 72 Cys + 155 Val + 65 Met + 109 Ile + 209 Leu + 175 Lys + 82 Phe + 575 Lac → Biomass + 23 Glu
**EM 42**	Cys → Amm + 3 CO_2_
**EM 43**	Glc → 6 CO_2_
**EM 45**	Lac → 3 CO_2_
**EM 62**	126 Glc + 311 Gln + 213 Ala + 215 Asp + 758 Ser + 114 Asn + 253 Gly + 56 His + 148 Thr + 153 Arg + 136 Pro + 67 Tyr + 72 Cys + 155 Val + 65 Met + 109 Ile + 209 Leu + 175 Lys + 82 Phe + 552 Glu → Biomass + 575 Amm
**EM 74**	Asp → Amm + 5 CO_2_
**EM 98**	442 Glc + 311 Gln + 213 Ala + 215 Asp + 758 Ser + 114 Asn + 253 Gly + 56 His + 148 Thr + 153 Arg + 136 Pro + 67 Tyr + 72 Cys + 155 Val + 65 Met + 109 Ile + 209 Leu + 175 Lys + 82 Phe + 552 Glu + 56 Amm → Biomass + 23 Glu
**EM 100**	126 Glc + 311 Gln + 213 Ala + 215 Asp + 758 Ser + 114 Asn + 253 Gly + 56 His + 148 Thr + 153 Arg + 136 Pro + 67 Tyr + 72 Cys + 155 Val + 65 Met + 109 Ile + 209 Leu + 175 Lys + 82 Phe + 632 Glu + 56 Amm → Biomass + 23 Glu
**EM 102**	Ser + Met → Amm + Glu + 3 CO_2_

The stoichiometry of EM macroscopic reactions considers only extracellular compounds, and is readily calculated as described in Methods. Some of these pathways involve only a single intracellular reaction able to operate in steady-state, whenever all the metabolites involved are also present extracellularly.

**Figure 3:**
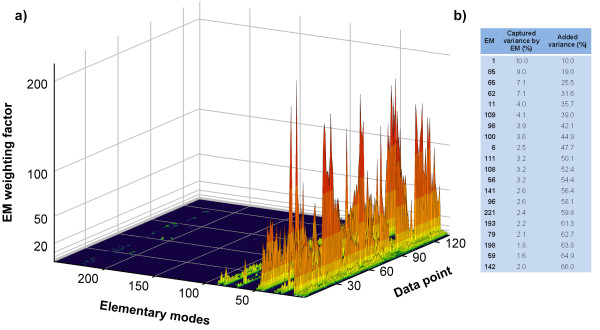
**Calculation of elementary modes weighting factors from flux vectors data (the same data of Figure 2) using the method proposed by Schwartz and Kanehisa **[29]. (**a**) Distribution of elementary modes weighting factor values among 134 flux data points and 251 elementary modes (**b**) Ranking of the 20 elementary modes with highest contribution to explained variance in flux data.

Several other methods were proposed to calculate EMs weighting factors from flux vectors but none of these methods use the correlation with the envirome as criterion for EMs discrimination. As pointed out by Wiback et al. [27], the extreme pathways (and elementary modes) weighting factors used to reconstruct a particular flux vector may not be unique. Linear programming methods can be applied to calculate the minimum and maximum values of such weighting factors, thus defining a range of possible values for each pathway, called the α-spectrum [27]. Unique solutions can also be obtained if additional assumptions about the biological system are considered. For instance, Nookaew et al. [28] proposed the determination of weighting factors by mixed integer linear programing to maximise the number of elementary modes that explain measured yield values. Using a different strategy, Schwartz and Kanehisa [29] proposed a quadratic program to minimize the sum of squares of weighting factors. To better illustrate our method, we compared it with the method proposed by Schwartz and Kanehisa [29], which basically selects the elementary modes that are closest to the actual biological sate by minimising the respective weighting factors. The application of this method to the 134 flux vectors data points identifies consistent subsets of 105 and 57 EMs with weighting factors bigger than 0.1 and 1.0 respectively (Figure 3a). As expected, this method selects a much higher number of EMs than PLP because it does not seek redundancy minimization. The 20 EMs with the highest contribution to flux data variance explained only 66% of variance (Figure 3b) compared to the 93% obtained with PLP (Figure 2a). This method also took 111 EMs to reach the 93% variance obtained with PLP with only 20 EMs. The 105 EMs with weighting factors bigger than 0,1 selected by the Schwartz and Kanehisa [29] method contain the 20 EMs selected by PLP but the former method does not link the EMs with the envirome.

On the whole, these results show that the most significant pathways in cultured mammalian cells could be identified from a larger, redundant set of possible routes using the correlation with the environmental state as the selection criterion. Further, the weighting factors of selected pathways are highly correlated with the environmental state denoting either a causal or effector relationship between environmental state and active pathways. Finally, such envirome-correlated pathways explain more than 90% of flux data variance.

### Assessment of pathway variability along culture time

Pathways may be up- or down-regulated either as a consequence of changes in the environment or driven by changes in the internal state ultimately reflected in a particular environmental "footprint". To monitor such regulatory processes, the PLP algorithm was applied to a moving time window, from the beginning to the end of each fed-batch experiment, to identify EM rearrangements over time. Each culture was partitioned into sequential metabolic phases, where *quasi *steady state is observed, according to the profiles of extracellular metabolite concentrations.

Table 2 illustrates the highly conserved active pathways obtained during growth in fed-batch 3 (for fed-batches 1 and 2, see Table 4 and 5, respectively). Again, a relatively small number of EMs was able to describe over 90% of the target rate data in every phase. A moderate degree of pathway conservation amid consecutive phases was observed, with, on average, 42% of the selected pathways appearing at least twice in each culture. This parallels with 38% of EMs repeatedly selected in at least two independent experiments (pooling together different phases within each culture), from which we can conclude that cells experience as much metabolic adaptation during culture as between cultures with different pre-set conditions. On the other hand, 65% of the selected EMs from the combination of all experiments in Figure 2b were conserved between cultures, showing the ability of the method to capture essential common features from independent data sets.

**Table 4:** Selection of active EMs during the growth for Fed-batch 1

Fed-Batch 1							
**Phase I**		**Phase II**		**Phase III**		**Phase IV**	

**EM**	**Var (%)**	**EM**	**Var (%)**	**EM**	**Var (%)**	**EM**	**Var (%)**

**20**	80.69	**53**	58.03	**62**	69.34	**62**	94.84
**2**	84.24	**1**	92.43	**1**	82.00	**1**	96.22
**-7**	87.54	**6**	93.65	**6**	88.68	**6**	97.51
**86**	90.79	**74**	94.76	**171**	92.55	**49**	98.49
**207**	91.81	**11**	95.54	**24**	94.38	**43**	99.21
**6**	92.79	**-4**	96.25	**43**	96.05	**24**	99.36
**1**	93.48	**26**	96.95	**7**	96.70	**26**	99.42
**11**	93.88	**102**	97.43	**-4**	97.01	**221**	99.46
**-5**	94.00	**2**	97.84	**102**	97.29	**7**	99.50
**102**	94.14	**220**	98.08	**5**	97.52	**87**	99.54
**3**	94.22	**41**	98.25	**2**	97.72	**11**	99.56
**9**	94.27	**7**	98.39	**11**	97.91	**4**	99.57
**-4**	94.31	**45**	98.45			**5**	99.57
**72**	94.34	**43**	98.50			**2**	99.58
		**-5**	98.52				
		**3**	98.55				

See Table 7 for time intervals.

**Table 5:** Selection of active EMs during the growth for Fed-batch 2

Fed-Batch 2									
**Phase I**		**Phase II**		**Phase III**		**Phase IV**		**Phase V**	

**EM**	**Var (%)**	**EM**	**Var (%)**	**EM**	**Var (%)**	**EM**	**Var (%)**	**EM**	**Var (%)**

**245**	61.90	**62**	92.01	**1**	82.42	**1**	29.01	**121**	34.08
**6**	77.86	**6**	95.85	**158**	95.65	**126**	56.64	**7**	51.99
**74**	82.59	**1**	97.69	**11**	97.11	**6**	79.18	**6**	64.06
**11**	86.59	**4**	98.40	**82**	97.72	**11**	85.38	**169**	73.55
**102**	88.74	**11**	99.07	**139**	98.24	**82**	89.67	**49**	80.98
**1**	90.68	**74**	99.30	**45**	98.33	**5**	91.12	**164**	83.33
**202**	91.67	**89**	99.33	**43**	98.40	**124**	92.88	**1**	84.78
**-5**	92.21	**220**	99.51	**-4**	98.48	**3**	94.21	**-4**	85.87
**86**	92.69	**7**	99.61	**5**	98.52	**4**	95.08	**5**	86.47
**41**	93.12	**43**	99.68	**42**	98.56	**43**	95.85	**183**	87.03
**4**	93.43	**227**	99.70	**3**	98.59	**7**	96.32	**3**	87.62
**87**	93.71	**45**	99.72	**7**	98.61	**181**	96.59	**11**	88.07
**43**	93.96	**14**	99.73	**6**	98.62	**24**	96.72	**44**	88.38
**7**	94.15	**24**	99.73	**24**	98.63	**112**	96.80	**120**	88.65
**14**	94.27			**88**	98.64	**102**	96.86	**9**	88.95
**15**	94.38					**45**	96.90	**225**	89.20
**45**	94.48					**15**	96.93		

See Table 7 for time intervals.

We further analyse in more detail the cases of the reversible EM 4 (Gln ↔ Glu + Amm) and EM 7 (Asn ↔ Amm + Asp), linked to amino acids metabolism, which were selected in all phases but at some point changed direction (the minus sign in Table 2). The main factors determining these changes in directionality were the limitation in glutamine concentration (EM 4 turned "negative") and the exhaustion of aspartate (EM 7 turned "positive"). The selection of such pathways (also repeatedly selected in all phases of fed-batch cultures 1 and 2) illustrates the ability of the method to tap on key metabolic adaptation processes.

### Consistency analysis of PLP reconstructions

**Figure 4:**
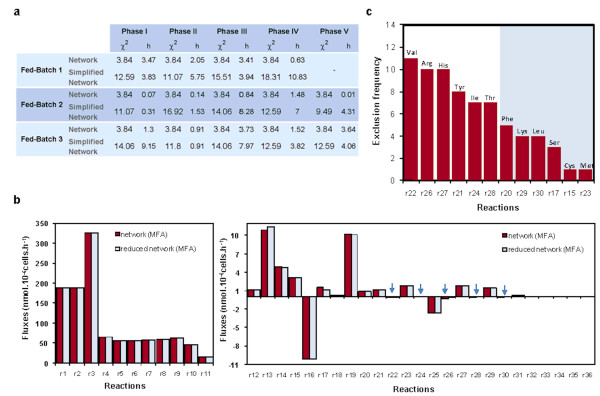
**Metabolic network reconstruction based on envirome data**. The selected EMs presented in Table 2 and 3 and in Additional File 1 were used to reconstruct reduced metabolic networks for each phase by excluding non-participating reactions. (**a**) Consistency analysis of metabolic flux estimations. The consistency index *h *Wang and Stephanopoulos [30], and the *χ*^*2 *^value (with degrees of freedom equal to the number of redundant measurements) are shown for original and simplified networks. Simplified networks have more degrees of freedom since excluding reactions is equivalent to adding null flux constraints. As shown, all phases have a *h *value lower than the corresponding *χ*^*2 *^value, meaning that the assumed biochemistry is consistent with the measured extracellular rates. (**b**) Intracellular flux estimations for the first metabolic phase of fed-batch 1. Excluded reactions are indicated with an arrow, showing null or negligible fluxes in the original network. See Figure 2b for an overview of all reactions and Table 6 for details. (**c**) Histogram showing the frequency of reaction exclusion in all metabolic phases of the three fed-batch cultures. These reactions correspond to the catabolism of some amino acids. The ones with low exclusion frequency should be controlled at stoichiometric levels in the medium to avoid ammonia accumulation. For the reactions excluded during each metabolic phase, see Table 6.

In order to assess the consistency of PLP results, the discriminated sets of EMs were back-transformed into reduced metabolic networks by excluding reactions not participating in any of the selected pathways. Then, metabolic flux analysis (MFA) was performed to compare intracellular flux distributions of the original BHK metabolic network and PLP reconstructed metabolic networks. Also the consistency index [30] was calculated for each stationary phase. The minimum number of EMs required for obtaining consistency between experimental data and the stoichiometry of simplified networks was the criterion to stop PLP reconstruction. This methodology yielded consistent networks with no more than 17 EMs (Figure 4a). Comparing the fluxes of original and reconstructed networks, it is clear that excluded reactions have fluxes close to zero in the original BHK network, while nonzero fluxes show almost imperceptible differences (for illustration, see Figure 4b with fed-batch 1 results).

The excluded reactions (actually pathways of lumped reactions) refer to the catabolism of amino acids (see Table 6), which normally occurs when their concentrations exceed the amount required for proteins synthesis [31]. Some are inactive during the majority of metabolic phases considered, namely r22, r26 and r27 (Figure 4c), corresponding to the catabolism of valine, arginine and histidine, respectively. Therefore, it can be concluded that the consumption of these amino acids is largely determined by protein synthesis (cellular and product), being practically unaffected by their extracellular concentrations within the range present in the medium. On the contrary, the consumption of phenylalanine, lysine, leucine, cysteine and methionine, (r20, r29, r30, r15 and r23, respectively) was largely influenced by their medium concentrations, since the corresponding catabolic pathways were active during more than half of the culture phases, suggesting these amino acids should be provided in controlled, stoichiometric amounts to minimize ammonia formation. A particular scenario arises for serine, whose catabolic conversion to pyruvate (r17) is down-regulated only during three metabolic phases (see Table 6). As mentioned, cells also convert serine into glycine for ammonia detoxification (r16 or EM 6): although maintaining a high extracellular concentration could be beneficial, it may also lead to net ammonia formation if present in excess.

**Table 6:** Excluded reactions by the PLP algorithm during each metabolic phase for fed-batches 1, 2 and 3

	Phase I	Phase II	Phase III	Phase IV	Phase V
**Fed-Batch 1**	22, 24, 26, 28, 30	17, 21, 22, 26	21, 22, 24, 26, 28, 29	15, 17, 20, 21, 22, 23, 26,28, 29	-
**Fed-Batch 2**	20, 22, 26, 27	17, 20, 26, 27	21, 22, 24, 26, 27, 28	22, 24, 27, 28, 30	21, 22, 27
**Fed-Batch 3**	21, 24, 27, 29, 30	20, 22, 24, 26, 27, 30	21, 22, 24, 26, 27, 28	21, 22, 26, 27	17, 20, 27, 28, 29

### Functional Enviromics Maps (FEM)

**Figure 5:**
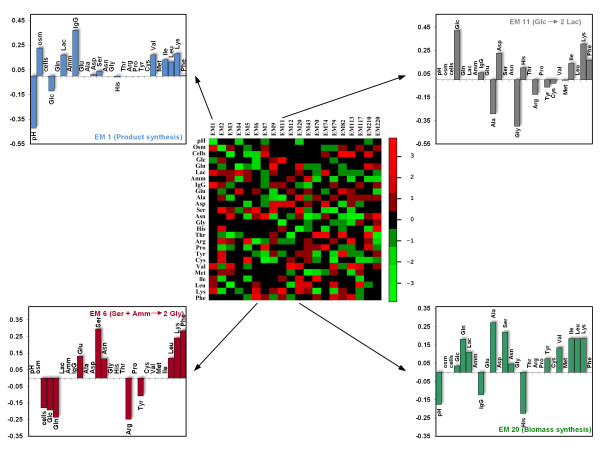
**Functional enviromics map obtained from the full envirome data set**. The regression coefficients of the PLP inner model were used to compute the effect of each environmental variable on the incremental variance in extracellular fluxes explained by each EM (columns in the colormap and vertical bars; see Figure 1 and Methods for calculation methods). Thus, they establish a statistical correlation between core cellular functions intensity and environmental factors, which can be exploited for the design of enhanced medium formulations and/or bioprocess operations.

Functional enviromics maps are intended to compile large-scale quantitative information of the interactions between environmental factors and cellular functions. A FEM consists of a data matrix, FEM = {I_i,j_}, with columns representing elementary flux modes, rows representing environmental factors, and I_i,j _elements the respective regression coefficients. In some cases, the magnitude of I_i,j _may be interpreted as the strength of up- or down-regulation of a given cellular function *i *by the environmental factor *j *(see Methods). The concept of FEM is illustrated in Figure 5 for the case of BHK cells using the full envirome data set, comprising the data of the 6 independent culture experiments.

Analysing these data, it can be observed that several envirome factors correlate positively with the flux for biomass synthesis through EM 20, namely the concentrations of glucose, lactate, glutamine and other 8 amino acids. On the other hand, extracellular pH and the concentrations of product and histidine correlate negatively. While glucose is known to be mandatory for many cell types in culture, BHK cells are capable of glutamine synthesis from glutamate [32]. However, it is also a major energy source for transformed mammalian cell lines, favouring growth when present in the medium. The remaining amino acids correlate positively with this EM since they are more concentrated during initial exponential growth when specific biomass formation is faster. As they are taken up by cells, the cellular division rate decreases as well.

In terms of product synthesis (EM 1), negatively correlated factors include the pH and glucose concentration, while osmolality and the concentrations of valine, isoleucine, leucine and lysine correlate positively with this flux. The negative correlation with glucose concentration is explained by bioreactor operation factors, as it decreases (or is maintained) along culture time whereas product formation rate increases, rather than by an inhibition mechanism on product synthesis. The effect of pH deserves further attention, since it also correlates negatively with biomass formation. As described in Methods, the bulk pH was allowed to vary between 7 and 7.25. Some reports agree that maximum growth rates [33] and recombinant protein productivities [33,34] are favoured at pH = 7, thus around the lower limit of the values used in this work. On the other hand, it has been shown that specific productivity in mammalian cells is higher with increased bulk osmolality [35-37]. Our results support this observation, however it should be noted that the increase in osmolality resulted mainly from the feeding of a concentrated solution during the fed-batch phase. As expected, glucose concentration positively correlated with the flux of lactate formation through EM 11, and serine concentration is the most important factor that activates glycine production through EM 6.

Despite using data from six independent experiments, some environmental factors (namely the concentrations of essential amino acids) show similar variation trends, which precludes disclosure of their true contribution to cellular phenotype. It is clear that the identifiability of environment-EMs relationships is conditional to sufficient "system excitation", which can only be assured by model-based design of experiments. Within proper circumstances of experimental design and physiological range of physical and biochemical variables, the information of EMs regression coefficients can be used to direct the phenotype to a desired state by manipulating genes, envirome or both. It should be noted that EMs represent clusters of genes, thus the EM regression coefficients translate the lumped effect of kinetic and genetic regulation induced by the envirome.

## Conclusions

While in functional genomics the aim is to unravel gene functions from the analysis of transcriptome and proteome dynamical data [38-40], here we propose a reverse, envirome-to-function analysis approach sourced by dynamic envirome data. This approach is founded on the assumption that the genome sets the universe of cellular functions while the envirome sets the relative contribution of each function to the observed phenotype. We have developed a systems level methodology that deconvolutes the observed phenotype (i.e. fluxome) into gene dependent structures (the structure of elementary flux modes) and envirome dependent structures (the relative weight of elementary flux modes ). Then, only the envirome dependent structures are linearly regressed against envirome components to discriminate the core cellular functions that correlate with the environmental state.

When applied to a recombinant BHK cell line, environmental data finds correspondence to less than 20 elementary flux modes, while explaining over 90% of flux variance. Most of the discriminated elementary flux modes are typical routes known to be active in cultured mammalian cells, while excluded routes are related with the catabolism of those amino acids not in excess in the medium, thus being directly used for protein synthesis. Furthermore, metabolic flux distributions of reduced metabolic networks, accounting for the discriminated core cellular functions only, were shown to be consistent with metabolic flux distributions of the original, unreduced metabolic network. These results essentially show that through the life of the culture, phenotypic variability is almost completely traceable by monitoring changes in envirome data. The remaining 10% of unexplained variance corresponds mostly to experimental error, thus leaving little room for genetically induced variability that cannot be traced to envirome data. On the whole, such results support the feasibility of a function reconstruction approach guided by the collection and analysis of envirome data sets.

When applied to a sliding time window along a single culture experiment, it was observed that several discriminated EMs are not active during all culture phases while others may change direction at some point. Such short-term dynamic changes correspond to metabolic adaptation induced by environmental stimuli. While some metabolic adaptation effects are readily interpretable in terms of exhaustion or limitation of essential substrates, some other have nontrivial interpretation and may correspond to unknown functions of environmental factors. The analysis of EM rearrangements can provide insights about the mechanisms underlying such metabolic adaptation. Despite the variability in observed EMs with culture time, a high degree of conservation of cellular function among the six different cultivation experiments was obtained (65% of discriminated EMs appear in all cultures). These observations support the results presented by Ma *et al*. [22] who used computational methods to identify network topologies that can achieve biochemical adaptation. They concluded that despite the diversity of possible biochemical networks, only a finite set of core topologies represent a robust adaptation response to a stimulus. These results support the hypothesis of a minimal set of core cellular functions activated by the envirome. The use of less informative envirome data sets (when compared to metabolome, proteome or transcriptome data sets) translates into lower discriminating power of cellular functions, particularly of those cellular functions producing similar environmental footprints. However, since only a small number of core cellular functions with distinctive footprints are active they are easily identified from an analysis of the envirome, thus constituting a vital support of cell functional enviromics.

While our intention was to demonstrate the principle of cell functional enviromics, its full potential can be realized when large-scale, high-throughput analysis techniques are employed for envirome-wide reconstruction of cellular functions eventually leading to high resolution functional enviromics maps. This approach has many interesting practical applications ranging from drug design to macroscopic process control. A point to be made is that finding a statistical correlation, even if linked with a metabolic structure, does not allow one to discriminate between a "cause" and an "effect". Such discrimination is only possible after a systematic analysis on the basis of a sound experimental design, which might include simultaneous environmental and genetic perturbations.

Fundamental issues, which remain to be clarified, include the degree of conservation of envirome functions among different species. One can anticipate that, given the role of environmental factors in the evolution of life, some degree of conservation may exist between species, as implicitly acknowledged by those who defend the reverse ecological principle [7]. A systematic functional enviromics study applied to different species and genotype variants could shed light on this issue.

The need for envirome-driven systems biology approaches to address human diseases has long been recognized [41]. Others have proposed enviromics as a complement to genomic and proteomic studies of mental health [42]. Even the notion of 'functional enviromics' has been set forth as a counterpart to functional genomics in tackling schizophrenia disorders [43]. Our approach has been to explore this concept in cellular systems- cell functional enviromics - which we view as a natural step in both fundamental and applied molecular biology research.

## Methods

### Envirome-guided projection to latent pathways (PLP)

According to the definition of elementary flux modes, the universe of infinite flux solutions, *r*, of a given metabolic network operating in steady state can be described as a non-negative weighted sum of a finite number of elementary flux modes, *e*_i _(e.g. [12,14,15]):(1)

Elementary modes thus represent unique and non-decomposable flux solutions, from which all possible system solutions can be obtained through the proper determination of elementary modes weighting factors, *λ*_i_. Several methods were proposed to calculate weighting factors, *λ*_*i*_, from flux vectors, r. (e.g. [27-29]). As noted by Wiback et al. [27], the reconstruction of a particular flux vector according to Eq. (1) is not unique if the number of elementary modes is higher than the number of fluxes, which is generally the case. Unique solutions can however be obtained with additional assumptions about the biological system, such as the maximisation of number of elementary modes [28], or minimisation of the values of weighting factors to select the elementary modes that are closest to the actual biological state [29]. In the present study we also calculate a unique solution for the weighting factors *λ*_*i *_although under completely different criteria. We set the weighting factors *λ*_*i *_as linear functions of envirome variables, x(t), which represents additional measured information in our method that is not used in other methods. The objective function in our case consists of:

i) maximising correlation between *λ*_*i *_and envirome variables (or equivalently between r(t) and x(t)), and

ii) minimising redundancy, i.e. eliminating all elementary modes with weak correlations with the envirome.

The basic idea is that the genome sets the structure of the elementary flux modes, while the relative weights of elementary flux modes are to a large extent set by the environment.

According to the above criteria, the PLP algorithm was designed to maximise the covariance between an input data matrix, X = {x(t)}, of independent envirome observations, x(t), and an output response matrix, R = {r(t)}, of observed steady state reaction rates, r(t), under the constraint of a plausible set of elementary flux modes. This problem can be expressed mathematically by the following constrained linear program:(2)

with X = {x(t)} a np × nx matrix of np independent observations of envirome vectors x(t) (dim(x) = nx), R = {r(t)} a np × nr matrix of np independent observations of reaction rates, r(t) (dim(r) = nr), E = {e_i_} a nr × nem matrix of nem elementary flux modes, e_i _(dim(e_i_) = nr) (for the BHK metabolic network, E is the 24 × 251 matrix given in the Additional File 1), Λ = {λ(t)} a np × nem matrix of weighting vectors, λ(t), of elementary flux modes (dim(λ) = nem) and *C *a nem × nx matrix of regression coefficients.

Maximising covariance implies two concomitant goals, namely maximising variance and minimising redundancy. Unconstrained maximisation of covariance can be achieved by the very popular projection to latent structures method, also known as partial least squares (PLS). Since PLP is derived from PLS, in the lines below we first review the structure of PLS and then show how it can be modified to PLP.

PLS is a multivariate linear regression technique that maximises the covariance between an input matrix, *X*, called the predictor, and an output response matrix, which here is the target rate data, *R*. It differs from traditional multivariate linear regression in that it decomposes iteratively both the predictor and the response matrices into a reduced set of uncorrelated *latent variables*, thereby eliminating redundancy in the input and output data sets. More specifically, the matrices *X *and *R *are decomposed into loadings matrices (*W *and *Q*), scores matrices (*T *and *U*) and residuals matrices (*E*_*X *_and *E*_*R*_):(3a)(3b)

The columns of the loadings matrices *W *and *Q *are the latent variables in which the input and output matrices are decomposed, respectively. Additionally, the outputs scores matrix U is linearly regressed against the inputs scores matrix T:(3c)

where *B *holds the regression coefficients determined by minimising the residuals *E*_*U*_. Finally, the explained variance in target data (which in our case is flux data, R) by the model is given by the following equation:(4)

with indexes *i *and *j *denoting observation and flux indexes respectively. Solving system Eqs. (3a-c), which imply minimising residuals E_X_. E_R _and E_U_, can be effectively performed by the very popular Non Iterative Partial Least Squares (NIPALS) algorithm. For more details about PLS and NIPALS see, for instance, Geladi and Kowalski [44].

PLP can be viewed as a constrained version of PLS by the inclusion of the elementary flux modes constraints. Given their equivalent mathematical structures, an interesting comparison may be made between projection of target reaction rate data, R, onto latent variables Q (performed in PLS according to Eq. (3b)), and projection of flux vectors onto elementary modes weighting factors according to Eq. (1). Indeed, elementary flux modes *e*_*i *_can be viewed as PLS latent variables equivalents, while the elementary modes weighting factors *λ*_*i *_can be interpreted as PLS latent variables score values. Following this analogy, a straightforward modification to Eq. (3b) can be introduced(5)

by substituting PLS loading matrix, Q, by the elementary modes matrix, E. This means that the latent structures *E *are no longer degrees of freedom in PLP as *Q *was in PLS. Instead, *E *represents a constraint to the relationship between X and R imposed by the metabolic network structure and ultimately by the genome of the cells.

An advantage of PLP over PLS is that both the latent variables and score values of the target matrix have in PLP a physical meaning. The latent variables are latent pathways while scores U are equivalent to Λ, i.e. they represent the relative weighting factors of latent pathways. For this reason, the algorithm is called projection to latent pathways. Moreover, the regression coefficients B can be used to deduce the functional enviromics matrix, FEM, as follows:(6)

FEM is a nx × nem matrix comprising the regression coefficients of elementary flux modes against envirome components, thus providing information of how elementary flux modes are up- or down-regulated by envirome components.

The PLP algorithm was coded in MATLAB (Mathworks, Inc) as a modified version of the NIPALS algorithm {Geladi, 1986 #15}, wherein the loadings of the outputs are fixed *a priori *according to the elementary modes structure, *E*. The calculation of the elementary modes is not automatically integrated in PLP. For that we used the METATOOL 5.0 [13] as explained below.

### Cell line and culture conditions

A BHK-21 cell line constitutively expressing the fusion glycoprotein IgG1-IL2 (antibody type one linked to Interleukin two) was used in this study [45]. Six cell cultures were performed in 2 L bioreactor vessels in a serum-free and protein-free medium, SFM4CHO (Hyclone), without glucose and glutamine. One culture was performed in batch mode and five in fed-batch mode. The starting volume was 950 ml, dissolved oxygen was kept at 30% of air saturation through air/nitrogen sparging at a gas flow rate of 0.02 vvm; the agitation rate was kept at 70 rpm. The pH was controlled between 7 and 7.25 by addition of NaOH (0.2 mM) or CO_2_. The batch culture started at 48 mM of glucose, 1.2 mM of Glutamine, 2.8 mM of Glutamate, 1.3 mM of Serine and 0.75 mM of Aspartate. As for the fed-batch cultures, the initial concentrations and feeding strategies are given in Table 1.

### Envirome profiling

27 envirome components were profiled, namely temperature, pH, osmolality and concentrations of 24 extracellular compounds determined as follows. Cell counts were determined in a Fuchs-Rosenthal haemocytometer; cell viability was assessed by the trypan blue dye exclusion method. Product concentration was determined by ELISA (see Teixeira *et al*. [46] for details). The concentrations of the main nutrients (glucose (Glc), glutamine (Gln), glutamate (Glu) and lactate (Lac)) were determined enzymatically using the biochemical analyser YSI 7100 (Yellow Springs, USA). The osmolality of culture supernatant was assayed using a Digital Micro-Osmometer, Type 5R (Hermann Roebling Messtechnik, Germany). Amino acids concentrations were determined by high performance liquid chromatography (HPLC) using a protocol optimized in our Lab (for details, see Carinhas *et al*. [47]). Ammonia concentrations were determined enzymatically using an UV assay (Boehringer Manheim, R-Biopharm AG, Germany).

### BHK Metabolic network

The BHK metabolic network adopted in this study comprised 57 metabolic reactions (including transport/diffusion across the plasma membrane), 35 intracellular metabolites and 24 extracellular metabolites and other compounds. Considered reactions explain the major fluxes of carbon and nitrogen, namely glycolysis, glutaminolysis, TCA cycle, pentose-phosphate pathway, recombinant product synthesis and biosynthesis of cellular components (nucleotides, carbohydrates, lipids and proteins). The catabolism of all amino acids except tryptophan is also considered. The network was simplified by lumping series of metabolic reactions, free of branching points, without loss of representativeness under steady-state. A detailed description can be found in Additional File 2.

### Metabolic flux analysis

For metabolic flux analysis (MFA), the difference between the number of reactions and the number of intracellular metabolites is 57-35 = 22, which means that at least 22 measured fluxes are required to obtain a determined MFA system. Envirome profiling provided data of 24 exchange fluxes, which means that the MFA system becomes redundant with 24-22 = 2 degrees of freedom. As such, the full vector of 57 fluxes was partitioned into known (extracellular) and unknown (intracellular) subsets of 24 and 33 fluxes respectively. Measured fluxes for different phases of fed-batch cultures are presented as Table 7. The resulting redundant system of linear equations was solved by the weighted least squares method [48]. The error standard deviation of measured fluxes were 5% for glucose, lactate, glutamine, glutamate and ammonia concentrations, 10% for biomass, product and the remaining amino acids concentrations. All MFA calculations were performed using the *FluxAnalyzer *software [13].

**Table 7:** Observed extracellular rates for several metabolic phases within three fed-batch cultures

	Fed-batch 1				Fed-batch 2					Fed-batch 3				
	
**R**_**obs**_	Ph I	Ph II	Ph III	Ph IV	Ph I	Ph II	Ph III	Ph IV	Ph V	Ph I	Ph II	Ph III	Ph IV	Ph V
	(21-45 h)	(45-70 h)	(70-95 h)	(95-118 h)	(25-50 h)	(50-70 h)	(70-90 h)	(90-110 h)	(110-135 h)	(50-75 h)	(75-100 h)	(100-125 h)	(125-150 h)	(150-175 h)
**μ**	0.019	0.022	0.022	0.012	0.020	0.020	0.018	0.014	0.009	0.020	0.017	0.014	0.009	0.008
**rGlc**	-190.33	-76.60	-71.99	-50.89	-187.25	-88.71	-74.53	-78.89	-51.89	-99.03	-71.38	-99.38	-63.53	-75.45
**rGln**	-8.74	-3.56	-5.24	-4.31	-10.23	-7.39	-2.02	-1.85	-1.20	-12.46	-3.73	-2.85	-1.47	-1.62
**rLac**	324.81	68.80	19.97	8.95	206.02	116.56	76.71	59.78	46.56	94.97	32.10	59.04	24.52	17.45
**rAmm**	9.85	12.94	10.50	9.68	16.32	12.75	9.88	8.17	6.34	9.83	9.40	12.91	14.04	12.47
**rIgG**	0.000215	0.000326	0.000291	0.000224	0.000167	0.000257	0.000301	0.000308	0.000264	0.000214	0.000280	0.000317	0.000368	0.000465
**rGlu**	3.90	-8.26	-15.43	-14.25	5.86	-9.97	-14.42	-18.72	-13.34	-3.49	-9.18	-12.19	-10.96	-13.61
**rAla**	0.13	-2.63	-2.83	-1.91	0.45	-2.34	-3.00	-2.27	-1.54	-1.65	-4.23	-4.16	-1.93	-0.56
**rAsp**	-15.89	-14.38	-8.20	-0.54	-12.71	-9.51	-2.22	-0.50	0.01	-5.96	-4.52	-4.34	-2.93	-2.33
**rSer**	-19.18	-16.59	-14.63	-3.06	-13.63	-10.89	-13.84	-14.43	-7.80	-15.58	-11.79	-10.03	-5.69	-4.47
**rAsn**	2.91	-3.98	-3.24	-2.31	-3.30	-3.05	-2.55	-1.86	-1.45	0.30	-2.37	-3.96	-2.96	-2.70
**rGly**	13.83	7.17	1.26	-0.01	9.50	7.34	6.18	5.84	5.16	8.53	6.30	3.66	1.93	0.47
**rHis**	-3.17	-2.80	-1.67	0.87	-1.08	-1.10	-0.65	-0.67	-0.47	-2.53	-0.78	-0.73	-0.52	-0.56
**rThr**	-3.30	-3.65	-3.47	-1.79	-2.99	-2.95	-2.53	-1.74	-1.30	-2.58	-2.10	-2.47	-1.37	-1.33
**rArg**	-3.15	-2.88	-3.38	-1.93	-2.88	-2.88	-2.55	-2.52	-1.41	-2.69	-2.35	-2.27	-1.47	-1.26
**rPro**	-5.84	-5.27	-1.81	-1.00	-5.82	-2.93	-0.83	0.90	1.60	9.34	2.41	-1.07	-0.39	-0.09
**rTyr**	-1.75	-1.35	-1.56	-1.13	-1.97	-1.41	-0.58	-0.39	-0.21	-1.17	-1.13	-1.02	-0.75	-1.08
**rCys**	-2.97	-2.58	-1.59	-0.88	-3.03	-1.45	-2.98	-3.34	-1.84	-3.36	-2.63	-2.54	-1.69	-1.41
**rVal**	-3.51	-3.84	-3.68	-2.04	-2.96	-2.82	-2.53	-2.20	-1.40	-2.56	-2.03	-2.50	-1.95	-1.77
**rMet**	-3.06	-4.76	-2.46	-0.72	-2.35	-1.89	-1.35	-0.82	-0.50	-1.68	-1.06	-1.46	-1.21	-1.32
**rIle**	-2.52	-3.16	-2.59	-1.54	-2.89	-2.40	-1.99	-1.41	-1.12	-1.93	-1.59	-1.68	-1.68	-1.47
**rLeu**	-4.66	-5.52	-5.11	-2.67	-5.01	-4.51	-3.47	-2.90	-1.85	-3.83	-3.07	-4.68	-3.29	-2.74
**rLys**	-5.62	-4.46	-4.27	-2.32	-5.25	-4.08	-3.35	-2.59	-2.07	-3.31	-3.62	-4.77	-2.96	-1.65
**rPhe**	-2.69	-2.27	-1.83	-0.75	-1.70	-1.60	-1.94	-3.25	-1.03	-0.98	-0.93	-0.98	-0.79	-0.80

Units are in nmol.(10^6^cells.h)^-1^. A minus sign indicates consumption. μ refers to the specific growth rate (h^-1^), calculated using the concentrations of viable cells

### Consistency analysis of metabolic flux distributions

Given that the MFA system is redundant with two degrees of freedom, the consistency index method [30] can be applied to verify consistency of calculated metabolic flux distributions in relation to assumed biochemistry and steady state assumption. Briefly, the consistency index, *h*, was calculated according to the method described in Wang and Stephanopoulos [30]. Then, the values of *h *were compared to the *χ*^2 ^statistical test for two degrees of freedom. Whenever *h *is below the *χ*^2 ^threshold value the system is said to be consistent. These tasks were performed using *FluxAnalyzer *[13].

### Determination of BHK elementary flux modes

The BHK metabolic network was firstly manipulated according to the guidelines in Gagneur and Klamt [49], and then the respective EMs were computed using *FluxAnalyzer *[13]. This medium scale network has a total of 251 EMs, which represent, in the context of the present study, the universe of cellular functions to be screened against envirome components. Among the full set of 251 EMs, 139 refer to biomass synthesis. A closer look to these EMs reveals that different by-products at different stoichiometric ratios are secreted or excreted concomitantly with biomass formation, namely ammonia, lactate, glutamate, alanine, aspartate and/or glycine, thus producing a distinctive environmental footprint that can be used for their discrimination. It should be noted that a single EM describes product synthesis since the underlying synthesis reaction involves only unbalanced amino acids (the carbohydrate content of the fusion glycoprotein was not considered). Additional File 1 provides a complete list of the 251 BHK elementary flux modes, formulated in terms of extracellular compounds stoichiometry.

### Application of PLP to BHK data

For the present BHK problem, the input data set X comprises the above enumerated 27 envirome factors. The target data-set R comprises measurements of the same 24 exchange fluxes used for MFA. The universe of EMs is formed by the 251 EMs obtained for BHK (see Additional File 1), The subset of EMs with significant correlation with the envirome is shown in Table 3.

## Authors' contributions

The project was conceived by RO and experiments planned by AT, RO, PA and MJTC. Experiments were performed by AT and NC. MS and AC supervised the bioreaction and JC developed software to support fed-batch control. PLP algorithm was developed by JD, MVS and RO. Data analysis and manuscript writing were performed by AT, JD, NC and RO. All authors read and approved the final manuscript.

## Supplementary Material

Additional file 1 **BHK elementary modes**. List of elementary modes obtained from the BHK metabolic network (additional file 2). Elementary modes are represented in reduced form in terms of extracellular metabolites. Click here for file

Additional file 2 **BHK metabolic network**. Biochemical reactions/pathways, enzymes and biomass composition considered in the metabolic model of BHK cells Click here for file
