# Supplementary material for: Cell functional enviromics: Unravelling the function of environmental factors
Source: BMC Syst Biol. 2011 Jun 6;5:92. doi: 10.1186/1752-0509-5-92 (PMC3118353; doi:10.1186/1752-0509-5-92)
Supplement: Additional file 2 — BHK metabolic network. Biochemical reactions/pathways, enzymes and biomass composition considered in the metabolic model of BHK cells [file 1752-0509-5-92-S2.DOC]

# Biochemical reactions, enzymes and biomass composition considered in the metabolic model of BHK cells

*Glycolysis*

R_1_: Glc ⭢ G6P (*Hexokinase*)

R_2_: G6P ⭢ 2 Pyr

R_3_: Pyr ⭤ Lac (*Lactate dehydrogenase*)

*TCA cycle*

R_4_: Pyr ⭢ AcoA + CO_2_ (*Pyruvate dehydrogenase*)

R_5_: ACoA + OAA ⭢ Cit (*Citrate synthase*)

R_6_: Cit ⭢ CO_2_ + α-keto

R_7_: α-keto ⭢ CO_2_ + SuCoA (*α-ketoglutarate dehydrogenase*)

R_8_: SuCoA ⭢ Fum

R_9_: Fum ⭢ Mal (*Fumarase*)

R_10_: Mal ⭢ OAA (*Malate dehydrogenase*)

R_11_: Mal ⭢ Pyr + CO_2_ (*Malic enzyme*)

*Glutaminolysis*

R_12_: Gln ⭤ Glu + Amm (*Glutaminase*)

R_13_: Glu ⭤ α-keto + Amm (*Glutamate dehydrogenase*)

*Amino acids catabolism*

R_14_: Pyr + Glu ⭤ Ala + α-keto (*Alanine aminotransferase*)

R_15_: Cys ⭢ Pyr + Amm

R_16_: Ser + CO_2_ + Amm ⭤ 2 Gly

R_17_: Ser ⭢ Pyr + Amm

R_18_: Asn ⭤ Asp + Amm

R_19_: Glu + OAA ⭤ α-keto + Asp (*Aspartate aminotransferase*)

R_20_: Phe ⭢ Tyr

R_21_: Tyr + α-keto ⭢ ACoA + CO_2_ + Glu + Fum

R_22_: Val + α-keto ⭢ 2 CO_2_ + Glu + SuCoA

R_23_: Ser + Met ⭢ SuCoA + Amm + Cys

R_24_: His ⭤ Glu + Amm

R_25_: Arg + α-keto ⭢ 2 Glu

R_26_: Glu ⭤ Pro

R_27_: Leu + 2 α-keto ⭢ 2 ACoA + CO_2_ + Glu

R_28_: Lys + 2 α-keto ⭢ 2 ACoA + 2 CO_2_ + 2 Glu

R_29_: Ile + α-keto ⭢ ACoA + CO_2_ + Glu + SuCoA

R_30_: Thr ⭢ ACoA + Gly

*Pentose-phosphate pathway*

R_31_: G6P ⭢ CO_2_ + R5P

*Cellular components synthesis*

R_32_: 26.5 Asp + 42.4 Gln + 5.3 Gly + 5.3 Ser + 10.6 R5P ⭢ 42.4 Glu + 15.9 Fum + DNA

R_33_: 67.8 Asp + 108.6 Gln + 27.1 Gly + 27.1 R5P ⭢ 108.6 Glu + 40.7 Fum + RNA

R_34_: 631.8 ACoA ⭢ Lipids

R_35_: 128.06 Glu + 213 Ala + 121.09 Asp + 177.89 Ser + 160.07 Gln + 113.6 Asn + 135.8 Pro + 220.5 Gly + 148 Thr + 175.3 Lys + 209.38 Leu + 108.95 Ile + 155.42 Val + 56.03 Met + 66.87 Tyr + 153.1 Arg + 72.29 Cys + 55.77 His + 82.36 Phe ⭢ Proteins

R_36_: DNA + RNA + Proteins + Lipids + 88 Glc ⭢ 10^9^ cells

*Product synthesis*

R_37_: 0.048 Glu + 0.034 Ala + 0.033 Asp + 0.073 Ser + 0.052 Gln + 0.035 Asn + 0.073 Pro + 0.028 Gly + 0.065 Thr + 0.081 Lys + 0.109 Leu + 0.037 Ile + 0.081 Val + 0.015 Met + 0.069 Tyr + 0.049 Arg + 0.016 Cys + 0.026 His + 0.065 Phe ⭢ IgG

Biomass composition

| Dry cell weight (DCW) | 390 pg/cell |
| --- | --- |
| Biomass composition  (weight percentage) | 72.9 % Proteins  13.5 % Lipids  1.4% DNA  3.5% RNA  3.5% carbohydrates |
| Number of DNA bases | 2 × (6.4×10^9^) |
| Average molecular weight of DNA bases | 309 g/mol |
| Average molecular weight of RNA bases | 325 g/mol |
| Average molecular weight of Fatty acids | 750 g/mol |
| Average molecular weight of amino acids | 110 g/mol |

The molar percentages of amino acids in cellular proteins were taken from .

References

1. Nyberg GB, Balcarcel RR, Follstad BD, Stephanopoulos G, Wang DIC: **Metabolism of peptide amino acids by Chinese hamster ovary cells grown in a complex medium**. *Biotechnol Bioeng* 1999, **62**(3):324-335.

2. Xie L, Wang DIC: **Applications of improved stoichiometric model in medium design and fed-batch cultivation of animal cells in bioreactor**. *Cytotechnology* 1994, **15**(1):17-29.

3. Zupke C, Stephanopoulos G: **Intracellular flux analysis in hybridomas using mass balances and in vitro 13C nmr**. *Biotechnol Bioeng* 1995, **45**(4):292-303.
